# West African Genetic Ancestry and Breast Cancer Outcomes Among Black Women

**DOI:** 10.1001/jamanetworkopen.2024.49798

**Published:** 2024-12-09

**Authors:** Sonya Reid, Run Fan, Lindsay Venton, Anne Weidner, Ann Tezak, Mya L. Roberson, Susan Vadaparampil, Xuefeng Wang, Sean Yoder, Marilin Rosa, Jibril Hirbo, Jennifer G. Whisenant, Jennifer Pietenpol, Padma Sheila Rajagopal, Brian Lehmann, Fei Ye, Tuya Pal

**Affiliations:** 1 Department of Medicine, Vanderbilt University Medical Center, Nashville, Tennessee; 2 Department of Biostatistics and Bioinformatics, Vanderbilt University Medical Center, Nashville, Tennessee; 3 Department of Health Policy and Management, University of North Carolina at Chapel Hill, Chapel Hill; 4 Moffitt Cancer Center, Tampa, Florida; 5 Department of Biochemistry, Vanderbilt University Medical Center, Nashville, Tennessee; 6 Center for Cancer Research, National Cancer Institute, Bethesda, Maryland

## Abstract

**Importance:**

Young Black women bear a disproportionate burden of breast cancer deaths compared with White women, yet they remain underrepresented in genomic studies.

**Objective:**

To evaluate the association of biological factors, including West African genetic ancestry, and nonbiological factors with disease-free survival (DFS) among young Black women with breast cancer.

**Design, Setting, and Participants:**

This observational cohort study included Black women diagnosed with invasive breast cancer between January 1, 2005, and December 31, 2016. Participants diagnosed with breast cancer at age 50 years or younger were recruited through the Florida and Tennessee state cancer registries. The final analysis was completed between June and September 2024.

**Exposure:**

West African genetic ancestry.

**Main Outcomes and Measures:**

A multivariable model was developed to evaluate the association between West African genetic ancestry and breast cancer DFS, adjusting for immunohistochemistry subtype, lymph node (LN) status, and full-time employment.

**Results:**

This study included 687 Black women with early-stage invasive breast cancer. Their median age at diagnosis was 44 years (IQR, 38-47 years), and the median follow-up was 10 years (IQR, 7-11 years). In multivariable analysis, triple-negative breast cancer (TNBC) and LN involvement were associated with shorter breast cancer DFS (hazard ratio, 1.81 [95% CI, 1.20-2.73] and 1.77 [95% CI, 1.30-2.41], respectively), whereas full-time employment was associated with improved outcomes (hazard ratio, 0.44 [95% CI, 0.30-0.63]). Among the 551 participants for whom global genetic ancestry could be assessed, having a higher percentage of West African genetic ancestry was associated with shorter breast cancer DFS among 246 participants in the hormone receptor (HR)–positive/human epidermal growth factor receptor 2 (*ERBB2* [formerly HER2])–negative subgroup (hazard ratio, 1.45 [95% CI, 1.04-2.04]). Of the 369 participants (53.7%) with PAM50 data available, basal (133 [36.0%]) and luminal B (107 [29.0%]) subtypes were the most common. Among the 179 patients with HR-positive/*ERBB2*-negative disease and PAM50 data available, luminal B and basal subtypes combined were also overrepresented (81 [45.3%] and 24 [13.4%], respectively) compared with luminal A (70 [39.1%]).

**Conclusions and Relevance:**

In this study of young Black women with breast cancer, having a higher percentage of West African genetic ancestry, TNBC, and LN involvement were associated with shorter breast cancer DFS. Interestingly, full-time employment was associated with improved breast cancer DFS. These findings highlight the importance of considering genetic ancestry beyond self-reported race and accounting for social determinants of health, in efforts to improve survival outcomes among Black women with breast cancer.

## Key Points

**Question:**

Is West African genetic ancestry associated with breast cancer survival?

**Findings:**

In this cohort study of 687 Black women with invasive breast cancer, West African genetic ancestry was associated with shorter disease-free survival, particularly among the hormone receptor (HR)–positive/human epidermal growth factor receptor 2 (*ERBB2* [formerly HER2])–negative subgroup. Moreover, tumor gene expression assays demonstrated overrepresentation of aggressive non–luminal A subtypes, which accounted for most tumors in the HR-positive/*ERBB2*-negative subgroup.

**Meaning:**

These findings suggest that inclusion of measured genetic ancestry, beyond self-reported race, and gene expression assays are important to improve prognostication of young Black women with breast cancer and address their disproportionate mortality burden.

## Teaser

This cohort study explores the association of West African genetic ancestry with disease-free survival among young Black women with breast cancer.

## Introduction

Breast cancer is the most common cancer type among women in the US and since 2019 has become the leading cause of cancer death among self-reported African American or Black (hereinafter, Black) women compared with White women, in whom it remains the second leading cause of cancer death.^1^ Although breast cancer survival rates have improved over the past several decades, Black women continue to have a 40% higher mortality rate compared with their White counterparts.^1^

Racial disparities in breast cancer survival are the result of both biological factors (eg, immunohistochemistry [IHC] subtype and tumor and germline genomics) and nonbiological factors (eg, social determinants of health [SDOH], patterns of care, and hormonal and lifestyle risk factors).^2,3,4^ Moreover, consequences of lower socioeconomic status (SES) and systemic racism contribute to worse survival.^5^ Even after controlling for SES, Black women with breast cancer still have a 30% higher mortality rate compared with their White counterparts.^6^ Taken together, these data suggest that although SES-related factors contribute to worse survival outcomes, they do not fully account for survival disparities observed in Black women.

Prior studies controlling for SES-related factors have consistently reported that Black race is a social construct and is associated with worse breast cancer outcomes,^7^ in part related to molecular tumor characteristics.^8^ Black women are more likely to be diagnosed with and die from breast cancer at younger ages compared with White women.^9,10,11,12,13,14,15,16,17^ Moreover, breast cancer diagnosed in Black women is more likely to demonstrate unfavorable characteristics associated with worse survival outcomes, including triple-negative breast cancer (TNBC) (an aggressive breast cancer subtype), larger tumor size, higher grade, and later stage at diagnosis.^2,18^

When evaluating survival disparities by IHC subtype, mortality rates appear similar between Black and White women with TNBC after adjusting for age, stage, and comorbidities.^19,20^ In contrast, studies using gene expression assays (specifically, prediction analysis of microarray 50 [PAM50]) among women with hormone receptor (HR)–positive breast cancer suggest an overrepresentation of non–luminal A subtypes in Black women compared with White women.^21,22,23,24,25,26^

Despite similar clinical and treatment patterns, Black patients with HR-positive/human epidermal growth factor receptor 2 (*ERBB2* [formerly HER2])–negative breast cancer have worse survival outcomes. This finding suggests, in part, a biological basis for the mortality gap among women with the HR-positive/*ERBB2*-negative subtype.^19,20,27,28,29,30,31,32,33^

Given the complexity in genomic backgrounds of admixed groups, use of self-reported race (a social construct) is a recognized limitation in biological studies of etiology and outcomes that use race as a subgroup or variable. Although a correlation exists between self-reported race and genetic ancestry, the latter is more robust when evaluating molecular tumor characteristics.^34^ In this study, our aim was to evaluate the association between West African genetic ancestry and breast cancer disease-free survival (DFS) while adjusting for potential confounders.

## Methods

### Study Cohort

Eligible participants in this cohort study were Black women diagnosed with invasive breast cancer at age 50 years or younger between January 1, 2005, and December 31, 2016, living in Florida or Tennessee at the time of diagnosis. Participants enrolled in the study within a median of 2 (range, 0-11) years of their breast cancer diagnosis. Upon approval by the institutional review boards at Vanderbilt University and the Departments of Health in Florida and Tennessee, registry-based recruitment was initiated. Per previously described state-mandated procedures,^35^ recruitment methods consisted of initial contact by mail and, if no response, a telephone call to explain the study and determine interest in participation. All participants provided written informed consent, and all research procedures were conducted in accordance with the Declaration of Helsinki.^36^ This study followed the Strengthening the Reporting of Observational Studies in Epidemiology (STROBE) reporting guideline.

### Clinical and Demographic Variables

Participants were asked to provide medical records, a release for tissue or tumor samples, and a saliva sample for DNA extraction. They were also asked to complete a study questionnaire, which included sociodemographic, epidemiologic, and lifestyle factors.

Data for all eligible participants who self-reported Black race, which included African American, Afro-Caribbean, and any multiracial category that included African American or Black, within the recruitment time frame were collected. Clinical data included age at diagnosis, stage at diagnosis, histologic subtype, IHC subtype, clinical stage and grade (using American Joint Committee on Cancer seventh edition staging), surgery type, chemotherapy, radiation therapy, endocrine therapy, and body mass index (BMI; calculated as weight in kilograms divided by height in meters squared) collected at the time of enrollment. Demographic data included county of residence, marital status, and primary payer at diagnosis. Data abstracted from medical records were supplemented with data provided from the state cancer registries and self-reported questionnaire data.

Information on IHC subtype of estrogen receptor (ER), progesterone receptor (PR), and *ERBB2* status was obtained based on medical record and pathology report abstraction, supplemented by cancer registry data and self-reported questionnaire data. Breast tumors were classified based on receptor status as follows: HR-positive (ER-positive, PR-positive, or both)/*ERBB2-*negative, HR-positive/*ERBB2*-positive, HR-negative/*ERBB2*-positive, and TNBC (ER-negative, PR-negative, and *ERBB2-*negative).

#### Primary Outcome

The primary outcome was breast cancer DFS, defined as the time from the date of diagnosis to the date of recurrence (local and regional or distant), death from any cause, or censored at the last follow-up date. Survival outcomes were collected from medical records, the TransUnion database, and follow-up data from the state cancer registries.

#### West African Genetic Ancestry

A saliva sample was collected from 601 participants using an Oragene self-collection kit (DNA Genotek Inc) and shipped to the investigators for DNA extraction. NanoDrop and Qubit technologies (both Thermo Fisher Scientific) were used for DNA quantification and quality assessment. DNA samples were stored at −80 °C prior to genotyping. A total of 376 samples were genotyped using Oncoarray (Illumina) and 175 samples were genotyped using the Multi-Ethnic Global Array (MEGA; Illumina). Standard sample- and variant-level quality control procedures were performed. Genetic ancestry proportions for each participant were estimated from multilocus single-nucleotide polymorphism genotype data using the maximum likelihood–based ADMIXTURE method^37^ in the R package *radmixture*, version 0.0.1, with the Globe13 calculator from the Dodecad Ancestry Project^38^ as a reference. The proportion of West African genetic ancestry was included in the multivariable analysis to capture potential population substructure.

### PAM50 Analysis

Through extracted RNA from formalin-fixed paraffin-embedded (FFPE) tumor tissue blocks, the PAM50-based breast cancer genomic signature was determined on the nCounter platform (NanoString) using the commercially available Prosigna assay (Veracyte). The PAM50 signature uses a combination of the level of expression of 50 target genes plus 8 constitutively expressed normalization genes to classify breast tumors into 4 distinct molecular subtypes (ie, luminal A, luminal B, *ERBB2* enriched, and basal-like). This test has been validated for use in FFPE samples,^39^ and all quality thresholds were applied automatically to the data by embedded software during the analysis process. For samples meeting all quality thresholds, a clinically validated algorithm was used to determine the molecular subtype, which is a prognostic indicator of the risk of distant recurrence of breast cancer.^40^

### Statistical Analysis

Continuous variables were summarized using medians and IQRs. Categorical variables were summarized using frequencies and percentages. A full multivariable Cox proportional hazards regression model of breast cancer DFS initially considered the following explanatory variables: West African genetic ancestry proportion, demographic factors (BMI and age at breast cancer diagnosis), SDOH (full-time employment, income, marriage status, education, and insurance), clinical factors (IHC subtype, stage, grade, histology, and lymph node [LN] status), treatment information (chemotherapy and radiation), and family history of breast cancer. Hierarchical cluster analysis on variables was performed to identify collinear factors and redundancy. Missing covariate data that could be reasonably assumed missing at random were imputed using multiple imputation (R package *mi*) with a predictive mean match to reduce bias and increase precision. Final models were developed using a backward selection procedure with a conservative α of .50 to avoid overfitting and enhance parsimony. Adjusted hazard ratios are reported with their corresponding 95% CIs. The R package *radmixture*, version 0.0.1,^37,41^ was used to estimate genetic ancestry proportions, including the percentage of West African individuals.

For participants with PAM50 data, the R package *genefu* was used for molecular subtype classification.^42,43^ Kaplan-Meier curves assessed DFS by PAM50 subtypes among the overall cohort and among participants in the HR-positive/*ERBB2*-negative subgroup. Statistical significance was set at *P* < .05 (2-sided). All statistical analyses were completed between June and September 2024 and were performed in R, version 4.2.3 (R Project for Statistical Computing) unless noted otherwise.

## Results

**Figure 1.**
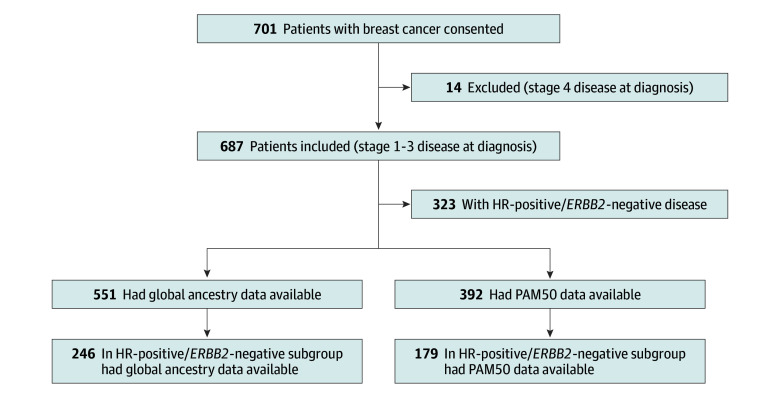
Study Schema *ERBB2* indicates human epidermal growth factor receptor 2 (formerly HER2); HR, hormone receptor; PAM50, prediction analysis of microarray 50.

**Figure 2.**
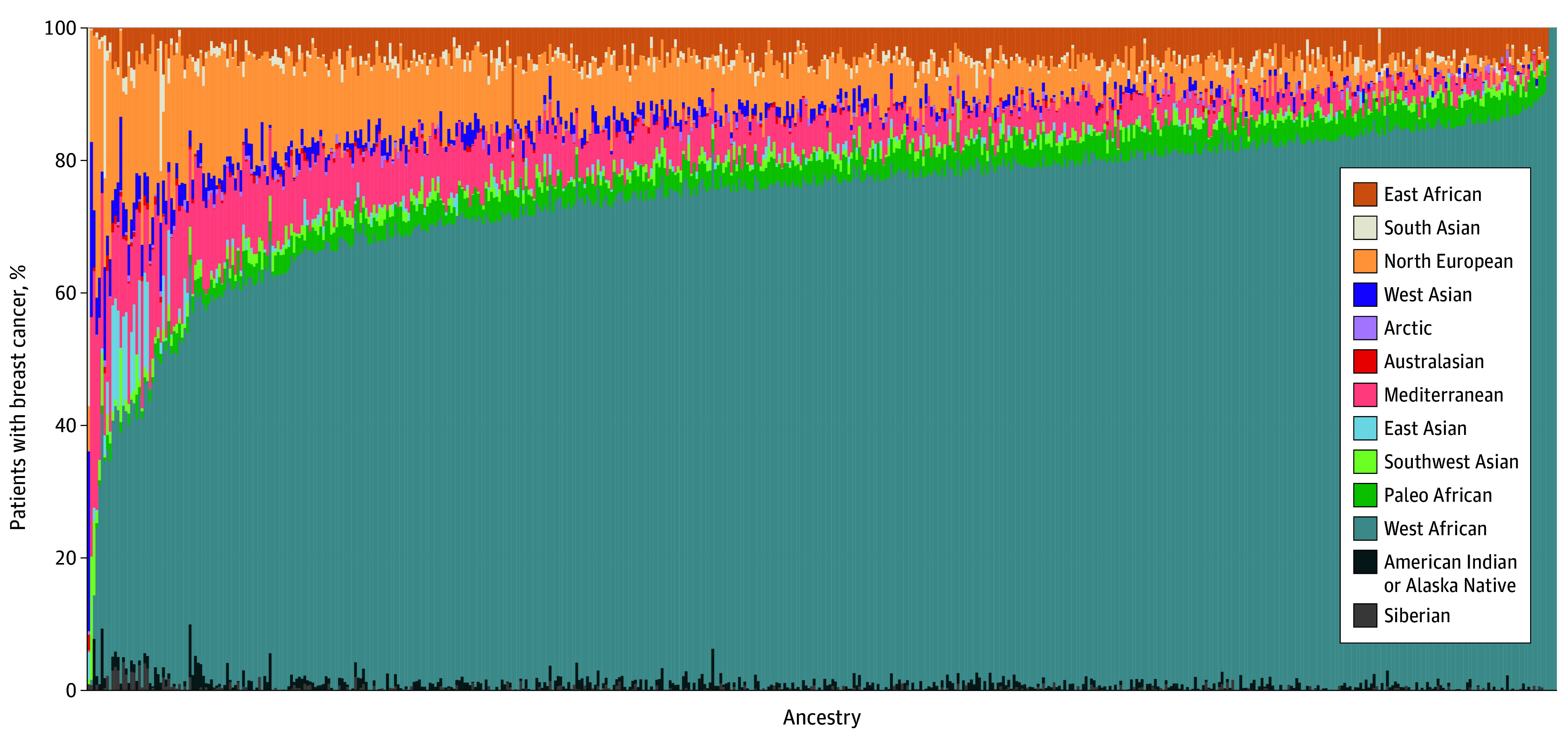
Global Genetic Ancestry of 551 Participants With Breast Cancer

Of the 701 patients who consented to participate, 687 were enrolled in the study. Their median age at diagnosis was 44 years (IQR, 38-47 years); 173 (25.2%) had TNBC, 263 (38.3%) had LN involvement, 390 (56.8%) had stage 2 or 3 disease at diagnosis, and 380 (55.3%) had grade 3 disease (Table 1). Fourteen participants with stage 4 disease at diagnosis were excluded from this analysis (Figure 1). Most participants (501 [72.9%]) received neoadjuvant or adjuvant chemotherapy and adjuvant radiation (445 [64.8%]). Of the 647 participants who self-reported a country of birth, 544 (84.1%) reported being born in the US, while the remaining 103 (15.9%) reported being born in the following countries: Antigua, Bahamas, Barbados, Dominican Republic, England, Germany, Grenada, Guyana, Haiti, Jamaica, Nigeria, Panama, Spain, Saint Christopher, Tanzania, Trinidad and Tobago, and Zimbabwe. Only 10 participants (1.5%) were reported by the state cancer registries to be of Hispanic ethnicity. Additionally, of the 574 participants with family history data available, only 14 (2.4%) reported their mother, father, or both as being Hispanic. Global genetic ancestry data were available for a subset of 551 participants, with a median West African genetic ancestry of 76.1% (range, 0.001%-100%) (Figure 2). More than half of participants were employed full-time (362 [52.7%]) and were insured (367 [53.4%]) (Table 1).

**Table 1.** Participant Characteristics^a^

Characteristic	Value (N = 687)
Age, median (IQR), y	44 (38-47)
Tumor size, cm	
Median (IQR)	2.0 (1.2-3.0)
≤2	218 (31.7)
>2	190 (27.7)
Missing	279 (40.6)
Lymph node status	
Positive	263 (38.3)
Negative	373 (54.3)
Missing	51 (7.4)
Stage	
1	263 (38.3)
2/3	390 (56.8)
Missing	34 (4.9)
Histology	
Ductal	572 (83.3)
Other^b^	97 (14.1)
Missing	18 (2.6)
Grade	
1	63 (9.2)
2	208 (30.3)
3	380 (55.3)
Missing	36 (5.2)
Immunohistochemistry distribution^c^	
HR-positive/*ERBB2*-negative	323 (47.0)
HR-positive/*ERBB2*-positive	76 (11.1)
HR-negative/*ERBB2*-positive	37 (5.4)
HR-negative/*ERBB2*-negative (TNBC)	173 (25.2)
Missing or *ERBB2* status unknown	78 (11.4)
BMI	
Median (IQR)	30.7 (26.4-36.5)
Missing	0
Surgery	
No surgery	10 (1.5)
Lumpectomy	255 (37.1)
Unilateral mastectomy	172 (25.0)
Unilateral mastectomy plus contralateral prophylactic mastectomy	217 (31.6)
Bilateral mastectomy	9 (1.3)
Surgery type missing	24 (3.5)
Chemotherapy	
Yes	501 (72.9)
No	158 (23.0)
Missing	28 (4.1)
Radiation	
Yes	445 (64.8)
No	238 (34.6)
Missing	4 (0.6)
Full-time employment	
Yes	362 (52.7)
No	275 (40.0)
Missing	50 (7.3)
Income, $	
<50 000	376 (54.7)
≥50 000	224 (32.6)
Missing	87 (12.7)
Insurance	
Insured^d^	367 (53.4)
Medicaid	129 (18.8)
Uninsured	41 (6.0)
Missing	150 (21.8)
Family history of breast cancer	
Yes	298 (43.4)
No	315 (45.9)
Missing	74 (10.8)
Education (college graduate)	
Yes	290 (42.2)
No	354 (51.5)
Missing	43 (6.3)
Marital status (cohabiting or married)	
Yes	271 (39.4)
No	374 (54.4)
Missing	42 (6.1)

Abbreviations: BMI, body mass index (calculated as weight in kilograms divided by height in meters squared); ER, estrogen receptor; *ERBB2*, human epidermal growth factor receptor 2 (formerly HER2); HR, hormone receptor; PR, progesterone receptor; TNBC, triple-negative breast cancer.

^a^ Data are presented as No. (%) of patients unless indicated otherwise.

^b^ Includes lobular/mixed ductal and lobular/other.

^c^ HR-positive includes ER-positive, PR-positive, or both. HR-negative includes ER-negative and PR-negative.

^d^ Includes private insurance, Medicare, and military insurance.

There were 102 all-cause deaths (14.8%) and 33 patients (4.8%) had recurrent disease, with 6273 person-years of follow-up and a median follow-up of 10 years (IQR, 7-11 years). In multivariable analysis, TNBC and LN involvement were associated with shorter breast cancer DFS (hazard ratio, 1.81 [95% CI, 1.20-2.73] and 1.77 [95% CI, 1.30-2.41], respectively), whereas full-time employment had a protective association (hazard ratio, 0.44 [95% CI, 0.30-0.63]) (Table 2). Among the 551 patients for whom global genetic ancestry data were available, having a higher percentage of West African genetic ancestry was associated with shorter breast cancer DFS among 246 patients in the HR-positive/*ERBB2*-negative subgroup compared with all patients with global ancestry data (hazard ratio, 1.45 [95% CI, 1.04-2.04]; *P* = .03 vs 1.23 [95% CI, 0.98-1.53]; *P* = .07) (Table 2). However, among 143 patients with TNBC and genetic ancestry data, West African genetic ancestry was not associated with breast cancer DFS (hazard ratio, 1.14 [95% CI, 0.80-1.63]; *P* = .48).

**Table 2.** Multivariable Regression Model

Variable	Adjusted hazard ratio (95% CI)	
	Overall cohort (N = 687)	Cohort with genetic ancestry data (n = 551)
Lymph node status		
Negative	1 [Reference]	1 [Reference]
Positive	1.77 (1.30-2.41)	1.83 (1.17-2.86)
Immunohistochemistry distribution^a^		
HR-positive/*ERBB2*-negative	1 [Reference]	1 [Reference]
HR-positive/*ERBB2*-positive	1.10 (0.62-1.97)	0.89 (0.43-1.84)
HR-negative/*ERBB2*-positive	0.89 (0.37-2.13)	0.94 (0.34-2.65)
HR-negative/*ERBB2*-negative (TNBC)	1.81 (1.20-2.73)	1.71 (1.07-2.74)
Full-time employment		
No	1 [Reference]	1 [Reference]
Yes	0.44 (0.30-0.63)	0.46 (0.29-0.72)
West African genetic ancestry	NA	1.23 (0.98-1.53)

Abbreviations: ER, estrogen receptor; *ERBB2*, human epidermal growth factor receptor 2 (formerly HER2); HR, hormone receptor; NA, not applicable; PR, progesterone receptor; TNBC, triple-negative breast cancer.

^a^ HR-positive includes ER-positive, PR-positive, or both. HR-negative includes ER-negative and PR-negative.

**Figure 3.**
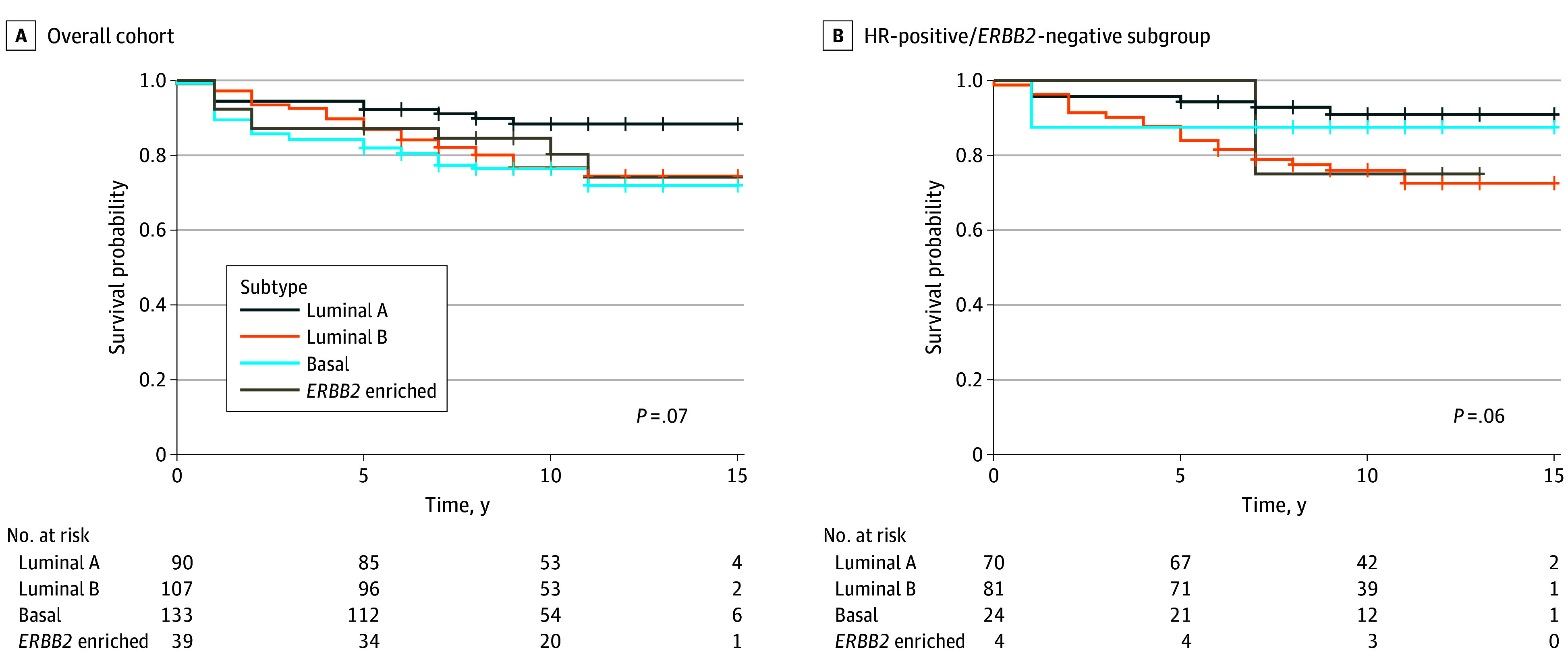
Kaplan-Meier Curves by Prediction Analysis of Microarray 50 (PAM50) Subtype A, Overall cohort. B, Hormone receptor (HR)–positive/human epidermal growth factor receptor 2 (*ERBB2* [formerly HER2])–negative cohort.

Of the 369 participants (53.7%) with PAM50 data available, luminal B (107 [29.0%]) and basal (11 [36.0%]) subtypes were the most common. Similarly, among the 179 participants in the HR-positive/*ERBB2*-negative subgroup with PAM50 data available, luminal B and basal subtypes combined remained overrepresented (81 [45.3%] and 24 [13.4%], respectively) compared with luminal A (70 [39.1%]) (eTable in Supplement 1). The Kaplan-Meier survival curves demonstrated shorter breast cancer DFS among participants with non–luminal A subtypes (ie, luminal B, basal, and *ERBB2* enriched) compared with those with the luminal A subtype, both in the overall cohort and in the HR-positive/*ERBB2*-negative subgroup (Figure 3; *P* values represent log-rank tests).

## Discussion

In this study, an association between West African genetic ancestry and breast cancer DFS among participants with HR-positive/*ERBB2*-negative breast cancer was observed, even after controlling for potential clinicopathologic and SDOH confounders. In addition, TNBC and LN involvement, known indicators of adverse prognosis, were associated with shorter breast cancer DFS in this study,^1^ whereas full-time employment was associated with longer breast cancer DFS. Finally, non–luminal A tumors were overrepresented among participants with PAM50 data available, which may, in part, account for the worse survival outcomes observed among Black women.

Although having a higher percentage of West African genetic ancestry was associated with shorter breast cancer DFS in this study, this finding was observed in the HR-positive/*ERBB2*-negative subgroup but not in the TNBC subgroup, suggesting that West African genetic ancestry may be differentially associated with survival outcomes based on IHC subtype. Consequently, our study highlights the importance of genetic ancestry–based analyses beyond self-reported race. To our knowledge, few studies to date have used African vs European genetic ancestry to evaluate differences in molecular and genomic tumor characteristics in the context of clinical outcomes.^34,44^ Among the few studies exploring genetic ancestry among Black survivors of breast cancer, having a higher percentage of African genetic ancestry was associated with developing TNBC, but not with outcomes after diagnosis of breast cancer.^28,45^ In contrast, our findings suggest an association between West African genetic ancestry and breast cancer DFS.

Interestingly, full-time employment was associated with longer breast cancer DFS in this study. Although employment status was one of several SES-related variables collected, several other variables exist that were not measured in this study (eg, financial considerations) but may further explain our findings. Prior studies have shown that a cancer diagnosis and cancer treatments have an adverse effect on employment, work ability, work performance, and work satisfaction among cancer survivors^46,47,48,49^ and higher mortality among working-age individuals who are unemployed.^50^ Furthermore, there is unsurprisingly a negative effect of cancer on employment, which in turn is a substantial contributor to financial toxicity.^51^ Although returning to work has been reported to be associated with a beneficial effect on survival among patients with oral cancer,^52^ lung cancer,^53^ and breast cancer^54^ in China, our study is the first (to our knowledge) to report that patients who were employed full-time had improved breast cancer DFS. The association we observed with employment may be related to several potential etiologies, such as benefiting from the socioeconomic advantages working may have afforded or feeling well enough to keep working after a cancer diagnosis.

We found an overrepresentation of aggressive basal tumors (133 patients [36.0%]), which substantially overlaps with the IHC-based aggressive TNBC subtype.^55,56^ These findings are consistent with prior studies reporting that proportions of molecular subtype differ across racial groups and contribute to survival disparities, with overrepresentation of the aggressive basal subtypes among Black women compared with White women.^27^ Moreover, in the HR-positive/*ERBB2*-negative subgroup in this study, aggressive luminal B and basal subtypes, which are associated with shorter breast cancer DFS, constituted the majority of tumors (81 [45.3%] and 24 [13.4%], respectively), consistent with prior studies that also showed an overrepresentation of non–luminal A subtypes in Black women compared with White women based on self-reported race.^19,20,28,29,30,31^ Similarly, in our study, Kaplan-Meier survival curves showed shorter breast cancer DFS among participants with non–luminal A subtypes compared with the luminal A subtype, both in the overall cohort and in the HR-positive/*ERBB2*-negative subgroup.^27^ Luminal A tumors have a high expression of ER/PR and have the best prognosis due to increased sensitivity of these tumors to endocrine therapies and a naturally indolent course. Conversely, luminal B tumors have a lower expression of ER/PR, more aggressive clinical and biological features, and greater likelihood of later recurrences. Basal HR-positive tumors are thought to behave similar to TNBC, with a high expression of Ki67 that results in an excellent response to neoadjuvant chemotherapy, whereas *ERBB2*-enriched tumors have increased sensitivity to *ERBB2*-targeted therapies. Worse survival outcomes among women with non–luminal A tumors in our study and others^19,20,28,29,30,31^ suggest that these tumors may, in part, account for worse survival outcomes among Black women.

### Strengths and Limitations

Major strengths of our study include the incorporation of both biological and nonbiological factors to determine associations with breast cancer DFS among Black women. Furthermore, our cohort focused on Black women diagnosed with breast cancer at age 50 years or younger; available cohorts generally are not focused exclusively on young patients with breast cancer.

Despite these strengths, a few limitations must be considered. This study only included patients who consented to participate, which could result in selection bias.^57^ Furthermore, a retrospective registry-based enrollment could lead to survival bias. However, we previously compared our participants’ data with data provided on all eligible women in the sampling frame provided through the state cancer registry, and no notable differences in demographic and clinical variables were observed.^58^ The use of all-cause mortality rather than breast cancer–specific mortality is another limitation, which may be mitigated by the young age of our overall cohort. Additionally, this study was not adequately powered to robustly measure effect modification by IHC subtype among participants with genetic ancestry data; therefore, these results will need further validation in larger cohorts. The smaller sample size of the TNBC subgroup could also have limited our ability to detect a smaller association with breast cancer DFS. Although robust biological and nonbiological participant data were collected, subgroup analyses were limited by our sample size.

## Conclusions

The results of this cohort study suggest that having a higher percentage of West African genetic ancestry is associated with shorter breast cancer DFS among women with HR-positive/*ERBB2*-negative breast cancer. Our results highlight the importance of measured genetic ancestry beyond self-reported race, taking into account SDOH. Moreover, our findings emphasize the importance of gene expression assays beyond standard IHC classification to potentially improve prognostication for Black women, who bear a disproportionate burden of breast cancer deaths. Moving forward, it is critical to include genetic ancestry and gene expression assays to both expand our knowledge and improve clinical tools, such as prognostic and predictive assays, to address the disproportionate mortality burden faced by young Black women with breast cancer.

## Notes

Supplement 1. **eTable.** Prediction Analysis of Microarray 50 (PAM50) Distribution

Supplement 2.
Data Sharing Statement
