# Supplementary material for: West African Genetic Ancestry and Breast Cancer Outcomes Among Black Women
Source: JAMA Netw Open. 2024 Dec 9;7(12):e2449798. doi: 10.1001/jamanetworkopen.2024.49798 (PMC11629124; doi:10.1001/jamanetworkopen.2024.49798)
Supplement: Supplement 1. — eTable. Prediction Analysis of Microarray 50 (PAM50) Distribution [file jamanetwopen-e2449798-s001.pdf]

Supplementary Online Content

Reid S, Fan R, Venton L, et al. West African genetic ancestry and breast cancer outcomes among Black women. *JAMA Netw Open*. 2024;7(12):e2449798. doi:10.1001/jamanetworkopen.2024.49798

eTable. Prediction Analysis of Microarray 50 (PAM50) Distribution

This supplementary material has been provided by the authors to give readers additional information about their work.

© 2024 Reid S et al. *JAMA Network Open.*

eTable. Prediction Analysis of Microarray 50 (PAM50) Distribution

	Overall Cohort (n=369) N (%)	HR+,HER2- Cohort (n=179) N (%)
Luminal A	90 (24.4%)	70 (39.1%)
Luminal B	107 (29.0%)	81 (45.3%)
Basal	133 (36.0%)	24 (13.4%)
HER2-enriched	39 (10.6%)	4 (2.2%)

© 2024 Reid S et al. *JAMA Network Open.*
