# Supplementary material for: West African Genetic Ancestry and Breast Cancer Outcomes Among Black Women
Source: JAMA Netw Open. 2024 Dec 9;7(12):e2449798. doi: 10.1001/jamanetworkopen.2024.49798 (PMC11629124; doi:10.1001/jamanetworkopen.2024.49798)
Supplement: Supplement 2. — Data Sharing Statement [file jamanetwopen-e2449798-s002.pdf]

# Data Sharing Statement

Reid. West African Genetic Ancestry and Breast Cancer Outcomes Among Black Women.
*JAMA Netw Open*. Published December 09, 2024. doi:10.1001/jamanetworkopen.2024.49798

**Data**
**Data available:** Yes
**Data types:** Deidentified participant data
**How to access data:** The data underlying this article are available in dbGaP at https://www.ncbi.nlm.nih.gov/projects/gap/cgi-bin/study.cgi?study_id=phs003466.v1.p1 and can be accessed with accession number phs003466.v1.p1.
**When available:** beginning date: 11-01-2023

**Supporting Documents**
**Document types:** None

**Additional Information**
**Who can access the data:** Deidentified participant genomic data will be available to download from dbGap for breast cancer researchers. Other data/statistical code will be made available upon request. Interested parties should contact the corresponding author.
**Types of analyses:** Breast cancer researchers interested in analyzing genomic ancestry data.
**Mechanisms of data availability:** Interested researchers can download deidentified data from dbGap. For additional data, interested parties should contact the corresponding author.
